# Experiences of delivering and receiving mental healthcare in the acute hospital setting: a qualitative study

**DOI:** 10.1186/s12913-024-10662-4

**Published:** 2024-02-12

**Authors:** Daniel Romeu, Elspeth Guthrie, Sonia Saraiva, Carolyn Czoski-Murray, Jenny Hewison, Allan House

**Affiliations:** 1 Leeds Institute of Health Sciences, University of Leeds, Leeds, UK; 2 Leeds and York Partnership NHS Foundation Trust, Leeds, UK

**Keywords:** Liaison psychiatry, Mental health liaison services, Qualitative, Experiences of care

## Abstract

**Background:**

Recent investment in UK liaison psychiatry services has focused on expanding provision for acute and emergency referrals. Little is known about the experiences of users and providers of these services. The aim of this study was to explore the experiences of users of acute liaison mental health services (LMHS) and those of NHS staff working within LMHS or referring to LMHS. A secondary aim was to explore the potential impact of a one-hour service access target on service delivery.

**Methods:**

Cross-sectional qualitative study. Individual interviews were audio-recorded, transcribed verbatim and interpreted using framework analysis.

**Results:**

Service users reported mixed experiences of LMHS, with some reporting positive experiences and some reporting poor care. Most service users described the emergency department (ED) environment as extremely stressful and wished to be seen as quickly as possible. Staff described positive benefits of the one-hour access target but identified unintended consequences and trade-offs that affected other parts of the liaison service.

**Conclusions:**

The assessment and treatment of people who attend ED with mental health problems needs to improve and particular attention should be given to the stressful nature of the ED environment for those who are extremely agitated or distressed.

**Supplementary Information:**

The online version contains supplementary material available at 10.1186/s12913-024-10662-4.

## Background

The number of people attending emergency departments (EDs) in England has continued to rise, aside from the COVID-19 period. In 2019/20, there were 25.0 million ED attendances compared to 21.5 million in 2011/12^1^. In April 2022, waiting time performance in EDs was the worst recorded in modern data collections [1], and people with mental health problems had to wait substantially longer than those with physical health problems. Although mental health presentations decreased during lockdown, there was a bounce back post-lockdown with even greater numbers attending ED than before [2].

There are relatively few studies of people’s experiences of liaison mental health services (LMHS) in the UK. A recent internet survey of respondents’ experiences of LMHS in England showed that only 31% of service users found their contact with such services helpful [3]. Latent class analysis identified three types of experience; those who had a positive experience, those who reported a negative experience and those who were non-committal. Suggestions for improvement included the provision of a 24/7 service, reduced waiting times for assessment, and clearer communication about treatment or care post-assessment.

Prior studies of user satisfaction of LMHS in the UK have also been mixed [4]. One previous study which involved in-depth qualitative interviews with service users found that people complained about long waiting times before being able to access liaison services [5]. Some service users reported good experiences characterised by close collaboration between the service user and liaison practitioner whilst others described po or experiences.

In contrast with the UK, studies from Australia have reported positive service user experiences of LMHS with high levels of service user satisfaction [6–8]. In one study, service users reported timely access to being seen by a liaison practitioner and reported feeling listened to, understood and helped in a positive fashion, with an emphasis on problem solution [6].

All 170 hospitals in England with an ED now have at least a rudimentary LMHS [9]. These services have undergone substantial growth in the last seven years following significant investment from NHS England [10]. There has been particular expansion in acute services, and a “Core-24” service model has been developed, with staffing ratios based upon hospital size in terms of bed numbers [11]. These Core-24 teams usually consist of at least one liaison psychiatrist and several liaison mental health nurses. They focus on emergency work, providing 24-hour cover for EDs and acute ward referrals, with an emphasis on one-off assessments followed by signposting.

These new developments have been accompanied by rigorous performance targets for response times and throughput. In 2016, NHS guidance stated that a person experiencing a mental health crisis should receive a response from a LMHS within a maximum of 1 h of receipt of referral, and within 4 h the person should have received: “*a full biopsychosocial assessment if appropriate, and have an urgent and emergency mental health care plan in place, and as a minimum, be en route to their next location if geographically different, or have been accepted and scheduled for follow-up care by a responding service, or have been discharged because the crisis has resolved”* [11]. Further review of access standards for mental health services in 2021 maintained the 1-hour target [9], although waiting time targets for all patients attending EDs are under review due to consistent and increasing failures to meet them.

While many hospitals have benefitted from the introduction of Core-24, especially where there were no or only rudimentary services previously, other established liaison services have had to change or modify their ways of working to meet targets. In addition to acute cover, these established services previously offered lower volume, higher intensity work involving the assessment, treatment and co-management of patients with complex physical and mental health problems seen in either inpatient or outpatient settings.

We previously completed interviews with 73 NHS staff from 11 hospital trusts in England who were either LMHS staff or worked closely with them and found that interviewees most valued being able to spend time with patients to carry out therapeutic interventions [12]. Some staff provided continued treatment for patients admitted to acute hospitals over several weeks. For example, in one service mental health nurses regularly visiting older adult patients or those on stroke wards to provide encouragement with eating and rehabilitation, both vital components of ensuring recovery. Teams with psychologists, therapists or mental health nurses trained in specific interventions (like cognitive behavioural therapy) offered brief interventions while the patient was admitted to an acute hospital bed, or a follow-up appointment after discharge. Staff reported problems with continuity of care across the secondary-primary interface; a lack of mental health resources in primary care to support discharge; a lack of shared information systems; a disproportionate length of time spent recording information instead of face-to-face patient contact; and a lack of a shared vision of care. Similar issues were identified across different liaison service types.

The aim of the present study was to better understand the experiences of users and providers of LMHS, and to explore hospital staff’s experiences of the changes brought about by the NHS England’s investment in Core-24 and any impact on patient care. We were particularly interested in improving our understanding of the mechanisms and trade-offs involved in relation to meeting one key performance target, the one-hour response time set by NHS England for LMHS. Recent programme theory suggests that the imposition of such fixed targets may have unintended consequences for liaison services and other parts of the health care system [13].

## Methods

This work formed part of the first phase of a programme funded through the NIHR Health Services and Delivery Research scheme to evaluate the cost-effectiveness and efficiency of different configurations of liaison psychiatry services in England (LP-MAESTRO) [14]. The Consolidating Criteria for Reporting Qualitative Research (COREQ) guidelines [15] have been followed.

### Design

This was a cross-sectional qualitative study with service users of hospital-based LMHS and hospital staff with either experience of working in, or working closely with, LMHS.

### Setting and sample

Service users were recruited from two Northern cities in England. We aimed to recruit 8–10 service user participants and developed a purposive sampling frame to ensure maximum variation. Potential participants were approached by either LMHS staff to determine their interest, or by local service user organisations who were invited to identify participants for the project through their own contacts. Once consent to contact had been provided by the service users, they were contacted directly by a member of the research team, who explained the study and provided a study information sheet. The potential participant was given at least 48 h to decide whether to participate. All participants provided written informed consent and there were no dropouts. No relationship was established with participants prior to study commencement. Participants were not informed of any of the interviewers’ personal goals for conducting the research.

Hospital staff were recruited from two hospitals in Northern England, both with EDs and within the same city. A maximum-divergence sampling frame was developed to maximise diversity according to professional background, sub-specialism within the LMHS, clinical or managerial focus and whether liaison team member or referrer to the service. Overall, we planned to recruit 8–10 staff participants. All staff participants provided written informed consent and there were no dropouts.

### Data collection

#### Service users

Nine service users were individually interviewed using a semi-structured topic guide. The service user topic guide was developed for this study (LP MAESTRO) and not published elsewhere (see Additional file 1). It consisted of a list of key topic areas with open-ended questions and additional prompts covering the following areas: introductory questions identifying the contact the participant had had with acute care; experiences of the acute care received from acute hospital staff; accounts of care received from LMHS staff; and views on desirable changes and ways to achieve them. They were not asked specifically about Core-24 developments, as it was unlikely that they would be familiar with such policy and staffing changes. However, staffing and waiting times were included as part of the topic guide.

### Hospital Staff

Eight hospital staff were individually interviewed using a semi-structured topic guide. The hospital staff topic guide was adapted from an earlier topic guide used in the LP-MAESTRO study in relation to a previous investigation of liaison psychiatry and hospital staff experiences of liaison services [12]. The adapted topic guide, which focuses primarily on staff experiences of CORE-24 is provided in Additional file 2. The following key topic areas were covered: introductory questions about the staff member’s work history and the nature of their involvement with LMHS; experiences of LMHS prior to introduction of Core-24; description of any changes resulting from Core-24; impact of these changes on the service; impact on patient care; and views on how the service could be improved.

Interviews lasted 30–90 min and took place via telephone between September 2017 and February 2019. Participant interviews were conducted first, followed by interviews with hospital staff. With permission all interviews were audio-recorded and transcribed verbatim. There were no repeat interviews. Transcripts were not returned to participants for comment or correction. Only the interviewer and participant were present at each interview. No field notes were recorded.

Interviews were conducted by three researchers, all from the Leeds Institute of Health Sciences and qualified by experience and training (CCG, SS, EG). None were involved in the delivery of acute LMHS at the time of the study. EG is a female Professor of Psychological Medicine and Consultant Psychiatrist. CCM is a female Senior Research Fellow and SS is a female Research Fellow. EG had previously worked in an acute liaison mental health team and was generally supportive of the Core-24 developments prior to the study. Neither SS nor CCM had a priori views or identified biases. The form and content of the topic guides were developed in collaboration with people with personal experience of mental health problems and accessing LMHS.

### Data analysis

The semi-structured interviews were interpreted independently by DR and EG using framework analysis [16]. This is a qualitative method that is useful in research that has specific questions, a limited time frame and a pre-determined sample; it is therefore well-suited to applied policy research. First, DR and EG independently read all transcripts with the study’s aim in mind. Each then independently reviewed all transcripts line by line identifying relevant experiences, opinions, descriptions of incidents and emotions (codes). DR collated codes into a draft theoretical framework which was refined through discussion with EG. It became apparent to base several framework categories around the key areas of interest in the interview schedule as we wanted to be open to issues arising from the data. DR then matched the data to the provisional framework. Each example was independently included under one or more theme in the thematic chart by DR and EG who then met to resolve any disparities. In the final stage, findings were reviewed by AH. Relevant supporting quotations were then extracted from interview transcripts to illustrate each theme and sub-theme. Data from service users and staff were analysed separately but are presented together if relevant to the theme or sub-theme. Participants were not asked to provide feedback on the findings.

## Results

### Sample characteristics

Seventeen in-depth interviews were conducted, nine with LMHS users and eight with healthcare professionals. Service user participants consisted of 3 men and 6 women with varying age ranges (Table 1). Presenting problems included self-harm, psychosis, mania, long-term physical health problems and medically unexplained symptoms. The interviewed professionals were mental health liaison nurses (*n* = 3), consultant liaison psychiatrists (*n* = 2), general nurses (*n* = 2) and one consultant in emergency medicine.

**Table 1:** Demographic characteristics and presenting health problems of service user participants

Gender	Age range	Ethnicity	Physical and mental health problems
Male	50–59	White-British	Long term physical and mental health problems
Female	20–29	White-British	Unexplained physical symptoms
Female	30–39	White-British	Multiple complaints-frequent user of emergency services
Male	60–69	Afro-Caribbean heritage /British	Renal failure
Male	30–39	White British	Long term physical condition
Female	40–49	White British	Severe mental illness
Female	50–59	African	Long term physical condition and mental health problems
Female	30–39	White British	Frequent self-harm
Female	40–49	White British	Severe mental illness

### Main findings

Participants discussed a range of topics surrounding the provision and experience of mental healthcare in general hospitals. They illustrated the complexity involved in meeting mental health needs in this setting. Below we outline our findings in terms of themes and sub-themes that emerged from the interviews; the four themes and their constituent subthemes are summarised in Table 2. The staff topic guide included specific questions about Core-24 that were not included in the service users’ topic guide, so most of the sub-themes around the Core-24 service standard are only relevant to staff participants.

**Table 2:** Themes and sub-themes from framework analysis of interview transcripts

Theme	Subtheme
**The emergency department (ED)**	ED staff
	Physical environment (service users only)
	Appropriateness for mental health problems
	Desired characteristics
**Liaison mental health services (LMHS)**	LMHS staff
	Barriers to contact
	Desired characteristics
**Core-24 service standard**	1-hour wait
	Perceived benefits (staff only)
	Unintended consequences (staff only)
	Policymaker detachment (staff only)
**Stigma of mental illness**	Discrimination
	Mental-physical dichotomy

### Theme one: the emergency department (ED)

Healthcare professionals and service users discussed their views of the ED as a site for mental healthcare provision. The content of their discourse comprised the sub-themes of *ED staff, physical environment, appropriateness for mental health problems* and *desired characteristics.*

### ED staff

Service users recounted highly variable experiences of ED staff when seeking help for their mental health problems. Some were described as kind and compassionate people who acknowledged distress and evoked feelings of validation:

“*They recognised I wasn’t putting things on, that I did feel acutely suicidal as I was saying*” – Participant 1.

Others disclosed negative views of ED staff, describing them as unpleasant and harsh. Three participants reported that ED staff withheld treatment that they thought was needed. Others reported that staff did not allow the service user to speak and failed to provide any guidance or support on discharge.

*“The GP referred me to A&E and when I arrived there, they were very very harsh”* – Participant 6.

LMHS professionals generally had negative perceptions of ED staff, reporting that they had poor psychiatric knowledge and skills. Several felt that ED staff did not appreciate the role of LMHS and frequently made inappropriate referrals. Some suggested that ED staff had little interest in mental health problems:

*“I think sometimes they don’t ask more questions about mental health, and I don’t know if that is because they don’t feel confident to, or they just don’t want to”* – Participant 13.

### Physical environment

The ED environment was discussed exclusively by service users, and their opinions were overwhelmingly negative. Common issues were that the assessment room was uncomfortable and small:

*“You’re brought into this really small room with no windows, it was tiny, it was also not necessarily painted, it was very scruffy*” – Participant 5.

A lack of provision of refreshments contributed to a sense of discomfort. The privacy of the assessment environment varied; two individuals reported that they were assessed in a private space, and one was not:

*“In the department with just a curtain pulled around, so it wasn’t very private”* – Participant 1.

### Appropriateness for mental health problems

Both service users and providers questioned the appropriateness of the ED for people with mental health needs. No participants felt that the ED was an appropriate place for these needs.

*“A lot of them are quite vulnerable, and more at risk of accidental self-harm or sort of vulnerable from other people in the department and it’s A&E isn’t it, I wouldn’t it consider a very nice environment for people that are experiencing psychosis”* – Participant 13.

Service users described their experiences of seeking care in the ED as anxiety-provoking and lonely. They acknowledged that attending the department was an undesirable last resort, only done when other services and professionals could not be accessed in the community.

*“It’s not the best solution by any stretch of the imagination but, but it’s the only place that’s available” –* Participant 8.

### Desired characteristics

Participants suggested ways that the ED could be improved to better care for those with mental health needs. These included a more comfortable environment, the option to wait outside, better communication of next steps and knowledge of community-based support. One participant suggested the provision of company while awaiting input from the mental health team.

*“I don’t know what else they could do apart from have somebody sit with you all the time until the psychiatrist came or somebody to assess you”* – Participant 7.

Liaison practitioners also felt that the ED could be improved by providing more staff training in mental health assessments and improving referrals to the LMHS. This could reduce the volume of referrals and facilitate referral triage while reducing wait times for service users.

*“If you upskill the ED people to even basic then liaison psychiatry should be able to turn down referrals… And we have to remember in the middle of all of this is a patient”* – Participant 15.

### Theme two: Liaison mental health services (LMHS)

The second theme refers to participants’ views and experiences of liaison mental health services (LMHS). There are three sub-themes: *experiences of LMHS, barriers to contact* and *desired characteristics.*

### LMHS staff

Service users described variable experiences of the help they received from LMHS. Contact with LMHS helped some individuals to feel more comfortable and to understand the next steps. Some described a therapeutic benefit of talking in depth about their issues:

*“It helps me mental health, being able to talk about it and stuff” –* Participant 3.

Others voiced that LMHS were either unhelpful or contributed to them feeling worse. This was related to the feeling of not being listened to and the perception that no tangible help or support was offered.

“*I’ve not got time for them as they do nothing for me”* – Participant 9.

Some service users held the view that LMHS complete little more than a “box-ticking” exercise that offers little benefit to the service user. This was echoed by one of the physical healthcare professionals.

Common problems were that the professionals seemed rushed and incompetent. Three participants shared the view that LMHS staff were dismissive or disinterested. This led them to feel guilty and as though they had wasted the professional’s time.

*“Sometimes the mental health staff can be very dismissive and treat me like I’ve just wasted everybody’s time, and I should have just looked after myself at home” –* Participant 8.

Others described LMHS staff in a more positive light, reflecting that they allowed them to speak freely while listening carefully and acknowledging their needs. In some interviews, LMHS staff were described as caring and comforting. One participant felt that LMHS staff are underappreciated:

*“I know with my experiences with liaison psychiatry that they do a lot more than people may think”* – Participant 4.

Generally, interviewed professionals were complimentary towards LMHS staff, describing them as hard-working, knowledgeable, experienced, accessible, and committed to high-quality patient care. Participants had conflicting views on whether LMHS staff have a good relationship with the ward teams and whether they meet their expectations, although this was often attributed to a rise in demand for the service.

### Barriers to contact

Participants discussed barriers to accessing LMHS. Some service users recounted how input from LMHS was postponed or withheld because they were under the care of a community mental health team. This sometimes resulted in interactions with a “diversion team”, which was described as a frustrating, obstructive experience.

*“You just can’t get past diversion because they’ve been put in place to stop people like me who are known to the system… They’re basically there to go, ‘there, there, you’re ok, you go home and speak to your care coordinator tomorrow.’” –* Participant 8.

Staff felt that significant barriers to contact with LMHS included insufficient staffing levels, particularly out of hours, and a seemingly excessive amount of time completing documentation.

*“The [LMHS] team spend a long time writing things up and reporting… If we do make a referral for later in the evening or overnight, I don’t work nights, but they’re often told, ‘oh we can’t come and see the patient because we’re writing up our reports!’”* – Participant 12.

### Desired characteristics

Service users outlined factors that would improve their experience of receiving care from LMHS. Several participants described dissatisfaction with being discharged without a clear treatment plan and called for the provision of aftercare and more information about third sector organisations.

*“If someone’s self-harming or whatever they shouldn’t just be discharged. They need aftercare and everything. It should be in their care plan.”* – Participant 2.

Some described desirable characteristics of LMHS staff, which included compassion, knowledge, and clearer communication of delays and anticipated next steps. Service users expressed a desire to be treated as an individual and to be listened to attentively.

*“You need front-line staff who have the personal interactive skills to acknowledge, to offer comfort and explain what is going to happen, not front-line staff who make you more agitated or that they are confused”* – Participant 5.

Other desirable characteristics of the service identified include universal service provision across the country, a switch of focus from medications to psychosocial interventions, and a separate service for those who do not meet the criteria for admission but who feel unsafe to return home. Some service users voiced support for an acute mental health service separate from the ED.

### Theme three: core-24 service standard

This theme encapsulates views towards the Core-24 service standard and the subthemes are *the 1-hour wait, perceived benefits, unintended consequences*, and *policymaker detachment.*

### 1-hour wait

Although professionals acknowledged the importance of targets, many felt that the one-hour target was unattainable, particularly for those with complex presentations or substance issues. Some felt that it was inappropriate to assume that service users’ needs are constant throughout the day. There was a consensus that immediacy was prioritised over clinical importance, which manifests as brief introductions within the hour instead of careful, comprehensive assessments.

*“It’s not about how quickly you are seen, it’s about the quality of the interaction and I think if you are having to respond to patients in an hour that can sometimes compromise the quality”* – Participant 16.

In contrast, service users almost universally expressed a wish to receive contact from the LMHS as soon as possible, and even a one hour wait felt too long to wait if someone was very distressed.

*“When you are thinking of taking your own life, an hour is a lifetime”* – Participant 1.

#### Perceived benefits (staff only)

The most salient benefit reported by the healthcare professionals was investment in the LMHS. They described more financial investment into the service, and the creation of staff posts to expand the workforce, contributing to feelings of reassurance and comfort. Although participants acknowledged the associated challenges of training new staff, overall, this change was perceived as positive.

“The investment within the services has enabled us to, erm you know, to broaden out what we do” – Participant 17.

Generally, professionals explained that the service standard improved patient flow. They felt that one-hour reviews were conducive to faster discharges and the prevention of unnecessary hospital admissions. They also reported a greater focus on the service user experience and acknowledged the target as an opportunity to improve the service further.

*“I think that’s been a huge positive for the team because it’s made them think, actually, okay, we need to do this. How are we going to do it in the best way possible to get the service users experience and the standard of care for them as best as we can?”* – Participant 14.

### Unintended consequences (staff only)

Professionals also reported numerous undesired sequelae to the Core-24 one hour target. The first was that the target acted as an incentive for people to use the ED for their mental health needs in the knowledge that they would be seen quickly. This contributed to a rise in the clinical workload for both ED and LMHS staff.

*“It was an odd thing to do when you’re trying to decrease attendances, it’s like a bit of an incentive to [attend the ED]”* – Participant 11.

Some explained that the target had a detrimental impact on servicing providing ward cover, as LMHS staff are diverted from wards to the ED for initial reviews for new presentations. This results in delays on the wards and subsequently prolongs admissions.

*“They used to see people who were in the beds before the parvolex (a treatment following a paracetamol overdose) ended, but they’re just unable to do that now because of the amount of people in A&E to be seen”* – Participant 10.

The target has also had ramifications on working hours, with some participants reporting that their shifts were extended from eight to twelve hours, resulting in more lone working and reduced staff morale. This was identified as the reason for some staff members deciding to leave their jobs.

#### Policymaker detachment (staff only)

Generally, professionals felt that Core-24 was implemented poorly by policymakers and commissioners who were disconnected from the service. They described that no attempts were made to seek the views of clinicians, and that it was delivered as a compulsory change.

*“The way that this change was brought in was very top-down, there was very little engagement with the team”* – Participant 16.

One professional reported that they were informed with little notice that older people would be included in the remit of LMHS following the standard, and they received no formal training for this. The disconnect between policymakers and clinicians resulted in resentment among staff.

Service users echoed this idea by suggesting that policymakers were detached from the views and priorities of those seeking care. Some mentioned that these should be incorporated into decisions about LMHS provision:

*“I think that the service should get more involvement from the service user’s experience”* – Participant 6.

#### Theme four: stigma of mental illness

The final theme describes the stigma associated with mental health problems. The subthemes were *discrimination* and *the mental-physical dichotomy*.

#### Discrimination

Service users commonly felt discriminated against for having mental health problems. They described being treated differently to those with physical health problems, with their issues not being taken as seriously. Some recalled being dismissed and feeling guilty for accessing services.

*“If you’re physically ill that counts, it’s given a higher priority over mental illness”* – Participant 4.

Professionals also acknowledged the discrimination against those with mental health problems in the general hospital setting. They commented that service users with mental health needs are generally perceived as problematic and unwanted in the ED.

*“Patients with mental health difficulties in the emergency department are the difficult ones, the bad ones, the ones that upset the data, or the ones that don’t move out quick enough”* – Participant 15.

### The mental-physical dichotomy

This subtheme describes the clear delineation between physical and mental health in the context of healthcare services. Both professionals and service users commented that mental health needs are frequently neglected in physical healthcare settings. This is attributed to a perceived unwillingness to enquire about psychiatric symptoms and a tendency to ignore biopsychosocial determinants of health.

*“If I was to mention mental state, your consultants turn their faces away from me”* – Participant 9.

*“The traditional method of dealing with a lack of liaison psychiatry in the general hospital is to ignore the problem and just pretend it’s not there, to not notice that the patient is sad, not notice that they are anxious, to blame the patient, to discharge them early, to not take care of the wider side of psychosis difficulties that have prompted this admission”* – Participant 15.

Clinicians also perceived a divide between mental and physical healthcare professionals. Some LMHS staff felt that ED clinicians had poor psychiatric knowledge and skills, that they often made inappropriate referrals with minimal information, and that their service was not understood or appreciated.

*“I don’t think mental health is respected within the A&E department as a proper profession”* – Participant 13.

### Final analysis

The final stage of analysis is summarised in Table 3, which shows comparisons across the service user and staff groups whilst also reflecting the strength of the signals from the data (determined by the proportion of participants who voiced these opinions). It shows some striking differences in patterns but also several areas of agreement. The one-hour access target is seen differently by service users and staff whilst issues related to stigma are perceived as important by both groups.

**Table 3:** Data intensity mapping for key framework components and sub-components

		Service Users	Staff
**The emergency department (ED)**	ED staff are helpful and caring	*	-
	ED staff are unhelpful and dismissive	**	** (perception of non-ED staff)
	ED environment is stressful	***	*
	ED environment is not appropriate for people with mental health problems	***	***
	ED environment could be improved for people with mental health problems	**	**
**Liaison mental health services (LMHS)**	LMHS staff are helping and understanding	*	*** (perception of non-LMHS staff)
	LMHS staff are dismissive	**	-
	It is difficult to access LMHS	*	** (perception of non-LMHS staff)
	There are ways LMHS could be improved	**	**
**Core-24 service standard**	The one-hour access standard is the maximum time a person with mental health problems should wait in ED	***	*
	The one-hour access standard prioritises immediacy over clinical need and has unintended consequences for other parts of the liaison service	-	***
	Policymakers are detached from clinical services which results in poor implementation	-	**
**Stigma of mental illness**	People with mental health problems who attend the ED experience discrimination	**	**
	There is a mental-physical dichotomy in the acute hospital which prioritises physical health over mental health	**	**

–indicates no data present for this sub-component

*Indicates low intensity sub-component

**indicates medium intensity sub-component

***indicates high intensity sub-component

## Discussion

### How our results compare

There are relatively few qualitative studies of LMHS, so this study is an important addition to the field. The variable experiences of LMHS users in this study are similar to those described by Eales and colleagues [5] and consistent with the recently published online survey of LMHS users [3]; some people reported good treatment and care from LMHS, whilst others report poor care and an unhelpful experience. Despite the increased funding for LMHS in recent years, people’s experiences remain patchy and well below the satisfaction levels reported by users of services in Australia [7, 17–19]. However, it is difficult to compare services between countries with different healthcare systems.

Most service users felt that the ED environment contributed to additional stress and was an inappropriate place for people with acute mental health problems. This is consistent with previous studies [20, 21], which have highlighted the negative and stressful aspects of the ED for people with mental health issues and described the ED as overstimulating and lacking in comfort and privacy. This is set against a backdrop of a recent survey carried out by the Royal College of Psychiatrists which reported that more than three quarters of people referred to mental health services resort to using emergency services because their mental health deteriorates whilst waiting for an initial assessment [22].

### Wait-time targets

Opinions about the appropriateness and helpfulness of the one-hour performance target for LMHS varied between service users and staff. Service users highlighted the importance of being seen as quickly as possible in ED, particularly because the environment was stressful, but also because they were in a heightened state of distress and needed urgent relief. Some staff, however, believed the one-hour target distorted clinical practice with performance taking precedence over clinical need. This resulted in many unintended consequences including encouraging an increase in mental health ED attendances and a detrimental effect on other parts of the liaison service.

These findings support a logic model we previously developed to explain the impact an increase in liaison mental health provision may have on specific target response times [13]. Increased staffing levels initially enable LMHS to see more service users within the designated response target time, but various tensions and trade-offs within the system become apparent over time. If more service users attend ED due to the quicker response time, coupled with long waits in the community, pressure on the system increases again. This pressure causes a tension between the balance of ED work and the needs of patients with severe mental health problems who are inpatients in the acute hospital. The focus on ED and meeting the response target may result in potential disruptions to the care of hospital inpatients with deleterious clinical consequences and increased length of hospital stays. The introduction of a response target inevitably leads to unintended consequences in other parts of the healthcare system; the balance of advantages and disadvantages of the target across the whole system needs to be considered.

#### Stigma

Public stigma and discrimination against people with mental illness is not a new phenomenon and is still widespread in society [23]. A review of 42 studies of ED staff attitudes towards service users presenting with mental health problems, 14 of which were conducted in the UK, reported widespread perceived negativity, although positive experiences were also acknowledged [24]. The findings from our study suggests negative attitudes towards people with mental health problems are still problematic in the ED setting. A recent qualitative systematic review exploring stigma and discrimination experienced by mentally ill individuals seeking care for physical and mental health concerns suggests that stigma and discrimination significantly compromise the quality of healthcare relationships with services users [25].

#### What can be done?

The Royal College of Emergence Medicine has produced a useful toolkit for improving care of people with mental health problems whilst in the ED, which stresses that all people with either a physical or mental health problem should have access to ED staff that understand and can address their condition [21]. There is a clear driver from both the Royal College of Emergency Medicine and the Royal College of Psychiatrists to improve the care of people with mental health problems who attend ED. There has also been recognition of this problem by NHS England with funding in 2017–2018 of £18 million for 234 winter mental health schemes to help alleviate pressures in ED for people with mental health problems [26]. Most of the funding was allocated to mental health liaison schemes, community crisis resolution and discharge and step-down schemes. Although many individual schemes reported local positive benefits, there were no robust evaluations which would support national rollout of any of these schemes.

There is some evidence that small positive attitude changes towards people with mental illness can be achieved by specific stigma reduction interventions [27], although relatively few interventions have been evaluated in the ED. Most educational interventions have focused solely on knowledge acquisition for specific conditions such as substance misuse disorders [28]. However, the endemic nature of stigma towards mental illness suggests that multi-level changes are required at organisational and personal levels. The Lancet Commission on ending discrimination in mental health included a review of all forms of stigma and discrimination against people with mental health conditions in all settings and societies globally [23]. The authors made several recommendations including policy and societal changes and workplace changes. Of relevance to the ED setting, they recommend that all healthcare staff receive mandatory training on the needs and rights of people with mental conditions, co-delivered by people with lived experience of mental health issues.

The staffing recommendations for Core-24 LMHS were largely based upon the size of hospital and the knowledge that mental health issues account for 4% of ED attendances. However, recent work suggests a further 4% of ED attendances consist of people attending with a physical health problem but who also have significant mental health issues [2]. This suggests that current LMHS staffing levels need to be reviewed, as Core-24 guidance may have underestimated the workload demands on LMHS, and workload is better estimated by patient throughput than the size of the hospital in terms of bed numbers or the presumed percentage of people in ED who may require liaison services [29].

## Strengths and limitations

This study has several strengths. First, we met our recruitment targets, although, recruitment of service users took longer than we anticipated. There are no patient organisations that represent liaison service users, so recruitment can be challenging. However, we achieved a wide diversity of service user participants in terms of demographic characteristics and clinical problem areas. The most common clinical problems seen by liaison services in England are co-morbid physical and mental health problems, self-harm and cognitive problems [29]. Participants with co-morbid physical and mental health problems were represented in our participant sample. However, service users with cognitive problems were excluded from this study due to the inability to provide informed consent to participate. Second, the staff participants came from a range of professional backgrounds, including those who worked within LMHS and those who referred to LMHS. Third, we were able to explore both service user and staff perspectives about an important aspect of current service provision – the one-hour access target.

There were several limitations to the study. First, our sample size was relatively small and service user participants were only recruited from two geographical areas, and hospital staff from only two hospitals. We required members of staff who had experience of LMHS both prior to and subsequent to the introduction of Core-24, which limited the number of staff who we could interview and were willing to participate in the study. This also limited our ability to interview to the point of saturation. A larger staff sample may have resulted in other themes emerging so the findings of this study cannot therefore be generalised to other services in England, although many of the findings do accord with previous work in this area. Second, as discussed above, we were unable to recruit people with cognitive problems, making findings less relevant to liaison services for older adults. Third, interviews with participants and staff were conducted before the COVID-19 pandemic and its impact upon healthcare delivery. There was a marked drop-off in ED attendances during lockdown and the many liaison ED services were moved to other parts of the hospital to minimise spread of infection. Although there has been a clear bounce back in ED attendances among people with mental health problems post-pandemic [2], it is unclear to what extent services and service users have changed.

## Conclusions

This study provides compelling evidence that the assessment and treatment of people who attend ED with mental health problems needs to further improve. The negative staff attitudes described are unacceptable, services for aftercare following assessment are inadequate, and the immediate experience in ED is often negative.

Particular attention should also be given to the stressful nature of the ED environment for those who are agitated or distressed. It can be argued that the ED is not the most appropriate place for people with acute mental health needs, but at present, there is often no clear alternative. Diversion schemes are under development in some areas. However, there will always be a need for many people with mental health problems to attend ED, as people with mental health issues commonly also have physical health problems, which require investigation and management in parallel with their mental health difficulties. Whilst ED service users emphatically support the one-hour response target, the imposition of such targets can have unintended consequences on other parts of the liaison service which need to be balanced to ensure parity for LMHS users in ED and those admitted in the acute hospital as inpatients.

### Supplementary Information

**Additional file 1.**

**Additional file 2.**

## Data Availability

Data from this study are not available due to the qualitative nature of the study.

## Abbreviations

LMHS: Liaison Mental Health Service
ED: Emergency Department
