# Supplementary material for: Experiences of delivering and receiving mental healthcare in the acute hospital setting: a qualitative study
Source: BMC Health Serv Res. 2024 Feb 12;24:191. doi: 10.1186/s12913-024-10662-4 (PMC10860287; doi:10.1186/s12913-024-10662-4)
Supplement: Supplementary file 1 — Additional file 1. [file 12913_2024_10662_MOESM1_ESM.docx]

**Additional file 1.** Interview topic guide for service users.

**INTRODUCTION:**

(Suggested script). As part of your recent hospital attendance/admission, we are interested in your experiences of needing to see someone from mental health services when you are in a general hospital for a physical health problem. We want to talk to you about what happened to you, who you saw and whether this was helpful for your mental health problems. This is not meant to be about particular hospitals or mental health professionals that dealt with you, but we understand that you may want to talk about your own experiences and that might involve you having to do this.

You are welcome to tell your story as you want to, and the researcher may ask you about specific things or for a bit of explanation about something she does not understand.

We are interested in some specific issues about contact with mental health services. We might not ask you a lot of direct questions but use our topic guide as a prompt or to help us understand what you mean.

We also want to explore with you what services you would like to be available to people with mental health problems who also have physical health problems.

1. What name would you prefer me to call you?
2. Why did you attend hospital?

**EXPERIENCES OF CONTACT WITH ACUTE SERVICES**

1. How would you describe your experiences of contact with the emergency service?
2. Were you offered mental health support in the emergency department? Did you get offered other support or advocacy? Can you tell me about your experience?
3. At any point did you feel that the seeing the psychiatrists was necessary before the other staff would arrange your discharge?
4. Some people have said that they have been told that they would not be able to get medical treatment unless you saw the psychiatrist. Has anything like this happened to you? (Prompt: did anyone tell you that this was hospital policy?)
5. Did you understand why you were seeing the psychiatrist? Can you tell me about how this came about for you? (Prompt: do you know who requested the mental health team for you?)
6. If you were transferred to a ward or other department, did you understand what was happening?
7. Did you feel that there were misunderstandings between you and the hospital staff? Can you tell me more?

**EXPERIENCES OF CONTACT WITH LIAISON PSYCHIATRY SERVICES**

1. We are interested in the sorts of staff who are on duty for mental teams in the emergency department. Can you recall who you saw? Do you think it was a psychiatrist, a psychiatric nurse, a clinical psychologist? (Use examples of staff as prompts as required).
2. Were you still in the emergency department when you saw the (mental health professional – depending on answers above)?
3. Can you tell me about where you saw the psychiatrist (or other)? (Prompt: was the room/space private when you were given the assessment?)
4. Can you tell me a bit about what happened? For example, did you get asked questions about your mental state or your feelings?
5. Can you remember the sorts of questions you were asked? Are there any that stick in your mind? Why is that do you think?
6. Can you remember roughly how long this took?
7. Can you tell me if you think that the assessment was at the right time for you to be able to answer them properly? How would you have wanted this if it had to happen?
8. Did you feel that the staff understood you and what you needed? Were you comfortable and able to open up to them?
9. What happened to you after your treatment? Was it what you wanted or expected? Can you tell us a bit more about what you would have wanted?
10. Were you offered a further appointment as an outpatient after you left the hospital? Was this for mental health care or follow-up for your physical problem?
11. Were you given information about other help that you could get from other services (e.g., addiction, support groups, etc.)? Did you want this information?

**VIEWS ON DESIRABLE CHANGES**

Not all services are arranged in the same way across the country.

1. We would like to explore what you think are the most important things that should be available for patients (use prompt list if needed).
2. Have you any suggestions about what the most important things are for patients that you would like to see?
3. What general mental health support services do you think are needed in the NHS (in general hospitals and in community mental health services)?
