# Supplementary material for: Experiences of delivering and receiving mental healthcare in the acute hospital setting: a qualitative study
Source: BMC Health Serv Res. 2024 Feb 12;24:191. doi: 10.1186/s12913-024-10662-4 (PMC10860287; doi:10.1186/s12913-024-10662-4)
Supplement: Supplementary file 2 — Additional file 2. [file 12913_2024_10662_MOESM2_ESM.docx]

**Additional file 2.** Interview topic guide for hospital staff.

**INTRODUCTION**:

Introduce self and purpose of interview.

Explain voluntary nature and confidentiality/anonymity.

Ask for permission to audio-record interview, clarify timings, ask for any questions.

**BACKGROUND AND CONTEXT:**

1. Briefly explore professional background (what they do now, how long for, length of time working in current hospital/setting).
2. Explore their role in or in relation to the liaison psychiatry (LP) team, how they work for or with them (probe on: what they do day-to-day, length of time in that role/working with the LP team).
3. Map how they came to work for or with the LP service (probe on: how/when they first heard about LP, what previous experiences of LP involved, how they ended up in the current role).

**MAKE-UP OF THE CURRENT CORE-24 LP SERVICE (for LP practitioners only)**

1. Map different settings the member of hospital staff works in (EDs, wards, outpatient clinics, specialist services, other).
2. Identify characteristics of the LP service (probe on: whether there are separate teams for older and working age adults, how/who provides out of hours services). If the
3. Explore relationship the LP team has with overlapping services (probe on: any links with drug and alcohol services and psychology, whether/how the LP team refers to other services in hospital).
4. Map any non-clinical services provided (education, training, other non-clinical activities).

**DELIVERY OF CORE-24 (for LP Practitioners):**

Ask participants to provide illustrative examples throughout. Clarify that we do not want them to compromise patient confidentiality.

Explore each relevant clinical setting in turn: outpatient clinics, emergency department, non-urgent wards, specialist services. Explore clinical practice prior to the introduction of Core-24

Ask about experiences of the following clinical problems: repeat presentations in the emergency department after self-harm, dementia which does not explain the current admission, poor self-management of chronic conditions (e.g., diabetes), psychosis.

1. **Context**: explore what the LP team does in each setting (probe on: requests and referrals, types of patient, types of clinical problem).
2. **Actions**: explore what they do now and how they respond to different types of problem (probe on: processes and treatments available, how far responses vary and influencing factors, who delivers different assessments and treatments, availability of services and out of hours cover, whether LP teams refer onto other services, any duplication of services).
3. **Outcomes**: explore what happens because of their actions (probe on: patient re-admittance, patient health outcomes, length of stay, financial savings), what ‘ideal’ outcomes look like in this setting, how far these are achieved, what facilitates these outcomes, what are the barriers (probe on: resources, working relationships, systems/processes, other constraints), any examples of overcoming challenges.

**CORE-24 HISTORY AND ONGOING INFLUENCES:**

Some of the below will be more or less relevant dependent on the participant type (i.e. LP practitioner or hospital staff) with links to LP service) – start broad.

1. **Service history:** explore the introduction of Core-24 (probe on: when Core-24 was introduced, any additional funding or positions, main changes to staffing (ED/ward work balance), working practices, clinical focus, staff morale, any changes to the type of mental health problems they are asked to assess, coverage, response times in different settings, impacts, balance of ED to ward work if relevant etc.
2. **Positive impacts:** probe about any positive impacts, ask for clear examples (remind the interviewee to protect patient confidentiality).
3. **Negative impacts** probe for any negative impacts-ask for clear examples.

**FUTURE OF LIAISON PSYCHIATRY:**

1. **Local**: explore how they envisage their service in the future (probe on: whether they will stay the same or change, why, what do they think about this).
2. **National**: what do they think LP services should look like in the future (probe on: staff make-up, hours and days of coverage, acute pathway care only, RAID, outpatient clinics, therapy, Core and Core-24, enhanced and comprehensive).

**CONCLUSIONS:**

1. Is there anything else to add?
